# Topical injection of sclerosant to treat cheek subcutaneous venous malformation resulting in blindness: a case report

**DOI:** 10.1186/s12886-023-03128-4

**Published:** 2023-09-19

**Authors:** Lihong Cui, Xinyue Zhang, Li Xu

**Affiliations:** 1 Department of Ophthalmology, Shenyang Fourth People Hospital, Liaoning, China; 2 Department of Ophthalmology, Shengjing Hospital, Chinese Medical University, Liaoning, China

**Keywords:** Physician-compounded foam sclerotherapy, Vison loss, Subcutaneous venous malformation, Child, Rare disease

## Abstract

Loss of vision after topical injection of sclerosant is a rare and uncommon complication. We describe a case with vision loss following the facial injections of physician-compounded (PCP) foam sclerotherapy which was created by room air. A 3-year-old boy underwent injection of 5ml polidocanol foamed with room air to treat the venous malformation on the cheek near the left orbit. The boy experienced the whole facial swelling on day 2 after the third injection, especially involving the left side, the visual acuity in the left eye was counting fingers at 30 cm and the swelling reduced at 7 days later after referral. Fundus examination on day 15 revealed hemorrhage inferior to the optic disc and fluorescein angiography revealed blocked fluorescein. The OCT on day 15 showed the edema of the nerve fiber layer beside the fovea. The patient’s hearing was also impaired. PCP foam sclerotherapy with room air produced in typical concentrations, preparations as well as volumes always causes vision loss among children. Continued evaluation on the effects of product, gas, volume, and patient age identify optimal approaches will avoid the toxicity and side-effects caused by facial foam sclerotherapy.

## Introduction

It has been widely acknowledged that venous malformations (VMs) are rare developmental vascular disease consisting of ecstatic deficient in smooth muscle cells [1]. In the clinical treatment of superficial venous disease, physician-compounded (PCP) foam sclerotherapy has been considered to be effective and minimally invasive, during which process the sclerosing solutions have been applied in the treatment of varicose veins [2, 3], skin hemangiomas, telangiectasias and so on [4]. Among those sclerosants, the importance of polidocanol cannot be overemphasized, whose advantages are known for low toxicity, low incidence of allergy, and few severe complications were reported [5, 6]. However, there existed a paucity of contraindication or guidelines for the special patient group who are also vulnerable population-infants or children, and those have higher risk for complications.

Herein, we described a boy with cheek subcutaneous venous malformation near left orbit who was found to have a severe vision loss after intralesional injection of the sclerosant, polidocanol.

## Case Presentation

The parents of this boy approved and signed written informed consent for publication of this case report and all the accompanying images.

**Fig. 1:**
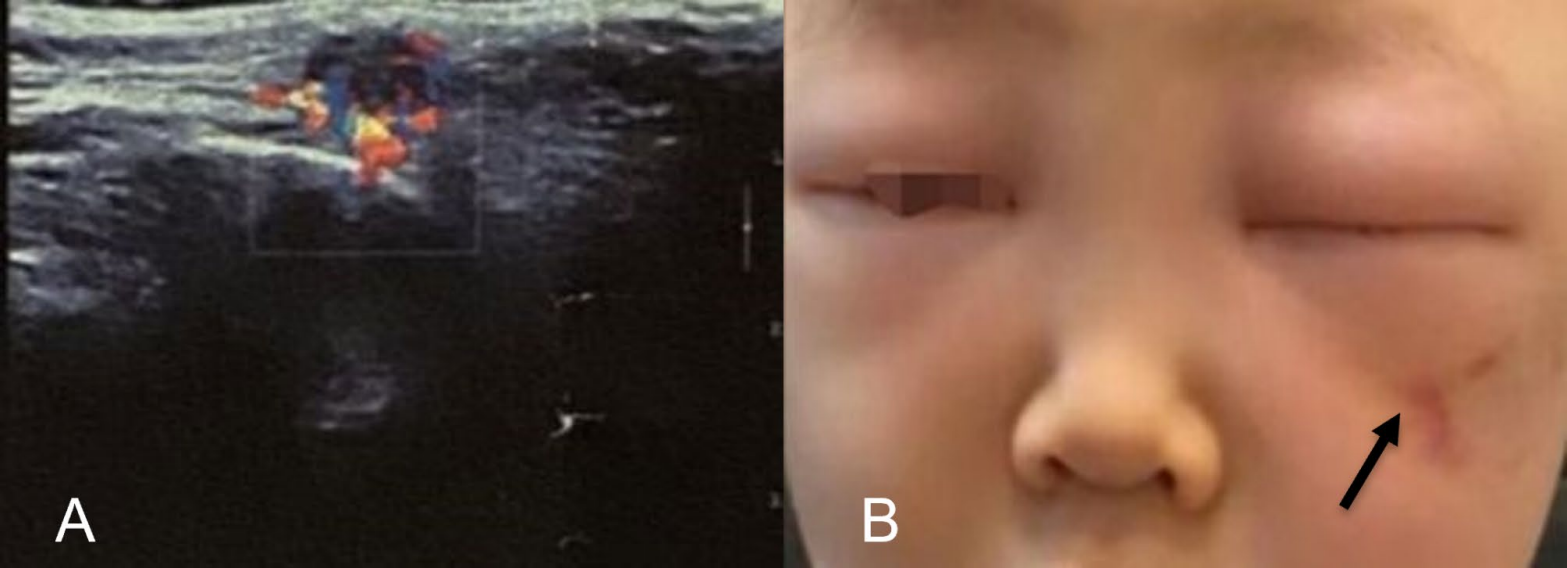
Subcutaneous B-scan showed a low-echo mass in the left cheek with the size of 1.6*0.8 cm (**A**). The venous malformation on the left cheek and facial swelling (**B**)

**Fig. 2:**
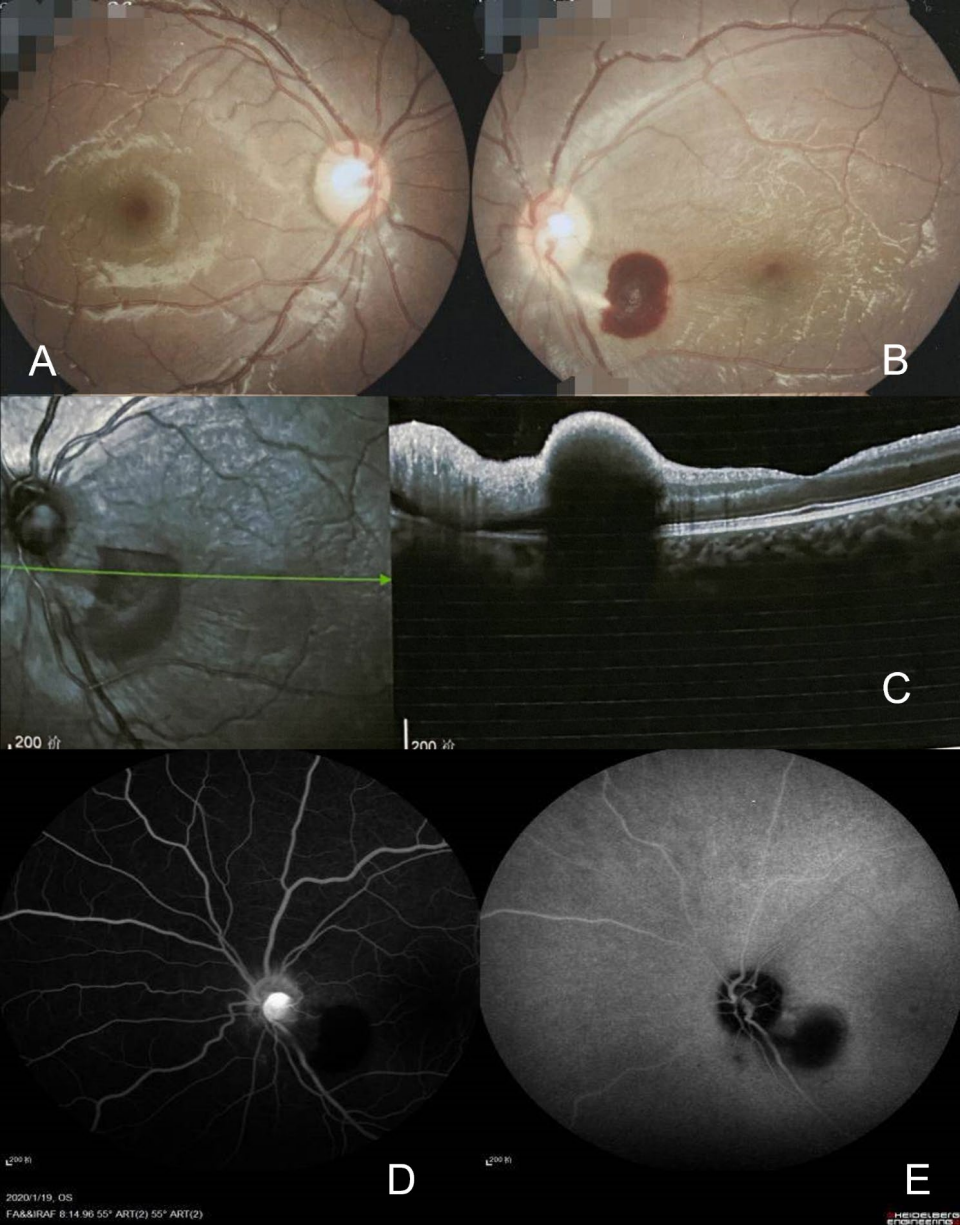
Binocular funduscopy. Right eye is normal (**A**); Hemorrhagic lesion on the infratemporal region of optic nerve head in left eye (**B**); The optical coherence tomography showed nerve fiber edema beside the macular (**C**); Fluorescein fundus angiography (**D**) and indocyanine green angiography (**E**) of the left eye showed hyperreflective leakage on the optic disk

A 3-year-old had a subcutaneous venous malformation of about 1.6*0.8 cm irregular area located at the inferolateral of the left cheek region. The boy developed a blue lesion on the skin gradually since one year ago and was finally diagnosed as venous malformation. In October, 2019, his mother was referred to radiologists for consultation. B-scan imaging at that time showed a low-echo mass in his left cheek (Fig. 1A). However, angiographic examination was not conducted. After that, the boy underwent percutaneous injection of 5 ml 3% polidocanol to the subcutaneous mass in his left cheek. Furthermore, 3% 5 ml foamed polidocanol injection mixed with 8 ml room air was conducted for treating venous malformation on the cheek by facial injection. The patient had had two injections under ultrasound visualization with 23-gauge needle tip by radiologists monthly. No abnormalities were found after the first two injections. However, after the third injection with the same dose of second injection, this boy began to cry loudly, and his left cheek developed redness immediately. On day 2 morning, the boy was found to manifest as swelling of the left eyelids and could not open his left eye (Fig. 1B). After three days, he experienced more unbearable pain with a more severe swelling involving the left cheek. Blood test indicated that his white cell count, C-reactive protein level and erythrocyte sedimentation rate (ESR) was 10 × 10^9/L, 1000 µg/L, and 15 mm/h, respectively. The patient was administered with Cefamezin and dexamethasone combined with hot compress daily for 4 days (day 3 to 6). On day 7, when the boy could finally open his eyes with the diminishing swelling, the visual acuity of the left eye declined to counting fingers at 30 cm, and band the visual acuity of right eye was normal. Unfortunately, hearing of his left ear was also impaired. Eye movements were normal. Bilateral intraocular pressures were 12/10 (right/left) mmHg. Fundus examination showed right eye was normal (Fig. 2A), and revealed a 1 papillary diameter (PD) hemorrhage located on the inferotemporal region of the optic disc, the margin of which was unclear, and the temporal veins were slightly expansive and tortuous (Fig. 2B). The optical coherence tomography (OCT) showed nerve fiber edema beside the macular fovea (Fig. 2C). Treatments was the same with day 3 to day 6. On day 17, Fundus fluoresce in angiography (FFA) and Indocyanine green angiography (ICGA) were performed with intravenous injection of half-dose fluorescein sodium and indocyanine green after sedation. FFA showed blocked fluorescence corresponding to the hemorrhagic lesion, high-reflective fluorescein leakage from optic nerve head at middle phase and slightly delayed ciliary artery filling time (Fig. 2D). There was reperfusion in the left retina at that time. The ICGA showed slow flow in the choroid of the left eye (Fig. 2E). The orbital magnetic resonance imaging (MRI) demonstrated that binocular position was inconsistent and there existed a soft tissue nodule located at the inferolateral of the orbital region with thickened Tenon’s capsule of the left eye. Furthermore, there were no orbital signs of compression of optic nerve. Moreover, the central vision of the left eye has lost, just remaining the peripheral vision. The possible underlying causes of left eye blindness and left ear deafness maybe extensive facial venous thrombosis extending to the left orbital and petrosal venous network due to over-ethusiastic sclerosant injection. However, the mechanism of injection resulting in blindness for this boy is still unclear because no pathological specimen can be obtained. At his last visit on 30 days, his BCVA was still counting fingers at 30 cm.

## Discussion

It is no doubt that sclerotherapy is a treatment to inject sclerosing solutions (sclerosants) into vascular abnormalities to induce localized thrombosis and eventually lead to fibrosis of the vessels. To the best of our knowledge, serious complications of foam sclerotherapy include anaphylactic reactions, cerebrovascular accident (CVA), transient ischemic attack (TIA), superficial venous thrombosis, tissue necrosis, edema, central retinal or posterior ciliary artery occlusion [7, 8]. Recent study indicated that the frequency of side-effects in patients receiving foam sclerotherapy to be approximately 0.9% [9]. Recently, with the development of sclerotherapy, many undesirable consequences also appear such as central retinal artery occlusion (RAO). Matsuo reported an 18-year-old patient afflicted with blindness, blepharoptosis, and total external ophthalmoplegia after sclerotherapy for glabellar subcutaneous hemangioma [10]. This complication might already appear on day 2 after injection but the boy was too little to describe, and the patient vision was poor as a consequence. Furthermore, Huang et al. found 10 cases presented RAO caused by cosmetic facial filler injections [11]. Moreover, experimental researches reported that subcutaneous injection of polidocanol may cause damage of muscle cells of the tunica intima and media of the blood vessels [12]. After balancing the pros and cons, facial injection of sclerosants should be more careful.

We postulated that the main causes of the vision loss could be summarized as follows: (1) sclerosants could flow into the fundus central artery or posterior ciliary artery from the facial primary lesion leading to obstruction and hemorrhage. But due to the fact that related ocular examinations were all performed about 2 weeks after sclerotherapy, the edema of retina, especially the fovea, could not be seen except the reperfusion lesion. (2) due to the MRI indication of thickened Tenon’s capsule, ocular tenonitis may exist, so it may be a chronic allergic reaction to sclerosants could lead to orbital tissue swelling, causing compression of these arteries. (3) the toxic reaction is also suspected because the boy was complicated with hearing impairment, but the FFA and ICGA did not indicate any vascular inflammatory reactions. It is known that the VMs have various channels which are intercommunicated, and the foam would spread to all areas. Thus, the sclerosant may transmit to retinal central artery through these channels.

In our case, the patient’s lesions of vascular malformation were located in the cheek subcutaneous area but not in the orbit. We cannot understand if the lesions had vascular connections with one another because no angiographic study was performed before treatment, and sclerosant might travel from the subcutaneous VMs to the central retinal artery. In our case, the patient did not have any complaints about the vision because of the swelling lid and the age. The patient also had hearing impairment caused by intralesionally injected sclerosant travelling from the subcutaneous Venous malformations to ear. On day 17, both FFA and ICGA showed almost normal experiences. These findings suggest that reperfusion might take place in the retina at some point, mimicking the condition of chronic retina ischemic presentation [13, 14].

Notably, image-guided sclerotherapy is becoming the preferred treatment for low-flow vascular malformations in head and neck region. However, it should be conducted under ultrasonographic and fluoroscopic guidance. Under this guidance, it would be safer in terms of control of volume of sclerosant required [15]. If embolism has occurred, anticoagulation is the main treatment method, such as oral anticoagulant Rivaroxaban, thrombolysis treatment and placement of intravenous filters if necessary [16]. Superficial thrombophlebitis (STP) is another complication of sclerotherapy in chronic venous disorders, with prevalence of 9.5% and common symptoms including pain, erythema, lumps, swelling around veins [17]. Using a blade or injection needle to remove blood clots in the affected limb’s blood vessels can quickly alleviate the patient’s symptoms. After treatment with foam sclerosing agent, hydrocolloid dressing and sodium aescinate can effectively improve STP symptoms. In this case, the exact site of injection sclerotherapy was close to the danger triangle of the face. The presence of networks of facial veins that are interconnected with skull base and intracranial veins. This is the basis of the concept of “danger triangle of the face” and the underlying reason of rapid extension of thrombosis and/or infect to the orbit and petrous.

In conclusion, sclerotherapy for cutaneous venous malformations on the left cheek near to the orbit might lead to extensive facial venous thrombosis extending to the left orbital and petrosal venous network due to over-ethusiastic sclerosant injection as a rare but severe side effects. More attentions should be paid to in patients with subcutaneous venous malformation during intralesional sclerosants injection.

## Data Availability

All data generated and analyzed during this study are included in this published article. Data and material are available from the corresponding author Li Xu (E-mail address: eyesiyuanyk@163.com).

## Abbreviations

OCT: optical coherence tomography
FFA: Fluorescein Fundus Angiography
ICGA: Indocyanine green angiography
MRI: magnetic resonance imaging
VM: venous malformation
PFO: patent foramenovale
CVA: cerebrovascular accident
TIA: transient ischemic attack
